# Effects of stigma, anxiety and depression, and uncertainty in illness on quality of life in patients with prostate cancer: a cross-sectional analysis

**DOI:** 10.1186/s40359-023-01159-6

**Published:** 2023-04-25

**Authors:** Shucheng Pan, Lijuan Wang, Li Zheng, Jie Luo, Jinjiao Mao, Wenbo Qiao, Binbin Zhu, Wei Wang

**Affiliations:** 1 Department of Nursing, the First Affiliated Hospital, Zhejiang University School of Medicine, Hangzhou, China; 2 Department of Urology, the First Affiliated Hospital, Zhejiang University School of Medicine, Hangzhou, China

**Keywords:** Social stigma, Quality of life, Anxiety, Depression, Prostate cancer, Effect analysis

## Abstract

**Purpose:**

Although much work has been carried out on stigma, anxiety and depression, and quality of life(QoL) in cancer patients, far less work has been done to examine their associations. This study explores the effects of stigma, anxiety and depression, and uncertainty in illness on QoL in prostate cancer patients.

**Methods:**

A cross-sectional study surveyed levels of stigma, anxiety and depression, QoL, and uncertainty in illness in 263 people diagnosed with prostate cancer from the First Affiliated Hospital, Zhejiang University School of Medicine. The main study variables were analyzed by structural equation modeling.

**Results:**

Anxiety and depression were significantly negatively related to QoL, with a standardized regression coefficient (*β*=−0.312, *S.E.* =0.478, *p* < 0.05), which means that participants reporting higher levels of anxiety reported decreased levels of QoL. Stigma was positively related to anxiety and depression (*β* = 0.135, *S.E.* =0.203, *p* < 0.001) and uncertainty in illness (*β* = 0.126, *S.E.* =2.194, *p* < 0.05). Stigma has direct effects on QoL (*β*=−0.209, *S.E.* =1.655, *p* < 0.001), but in the presence of a third variable (anxiety and depression overall), direct effects are reduced, as indirect effects emerge through the variable anxiety and depression overall, with an indirect effect size of − 0.054.

**Conclusions:**

Stigma impacts mental health, such as anxiety and depression, uncertainty in illness, and QoL. Health care professionals may help patients alleviate feelings of anxiety, depression, and uncertainty in illness to improve QoL outcomes.

## Introduction

Prostate cancer is the most frequently diagnosed malignancy in males in 112 countries, and it ranks second after lung cancer worldwide [1]. The incidence and mortality rates of prostate cancer are strongly related to age, with the highest incidence in elderly males [2]. The burden of prostate cancer is anticipated to continue to rise over time owing to population aging and an increasing life expectancy [3, 4].

Traditional therapeutic trials have largely focused on physiological outcomes and organ functioning. Over time, the field of medicine has recognized the relevance of both "the patient’s perception and the psyche as an overt contributor" to the cause and treatment of disease [5]. The term "health-related quality of life (HRQoL)" acknowledges that quality of life (QoL) includes many dimensions and refers to the "physical, psychological, and social domains of health, seen as distinct areas that are influenced by a person’s experiences, beliefs, expectations, and perceptions" [6]. QoL reflects an individual’s conscious and cognitive judgment of satisfaction with one’s life. A large population-based patient-reported outcomes study [7] on QoL demonstrated that patients with prostate cancer suffer from problems with sexual dysfunction, urinary difficulties, and vitality and hormonal issues. These long-term side effects, adding to the lack of helpful interventions and support, such as medications, devices, or specialist services, potentially impact their QoL [7–9].

A number of prostate cancer survivors experience invisible stigma [10]. Following Goffman [11], stigma was defined as a process in which society assigns attributes that mark an individual as different and undesirable, which can lead to reduced self-identity and social exclusion. Goffman’s theory of stigmatization describes that the subject’s health status can be affected due to the internalization of stigma, even if subjects do not suffer obvious unfair treatment. Studies have shown [12–14] that stigma stems from increased stress and poor coping, leading to negative mental health outcomes and the amplification of psychosocial distress. In general, cancer has been perceived as a highly stigmatized condition, ranging from 13 to 80% [15], often in that it is associated with death, changes in physical appearance and body image, social isolation, or blame and shame. Body image change by chemotherapy and androgen deprivation therapy (ADT) [16], feelings of loss of masculinity by sexual difficulties [17], and lower self-esteem mixed with stigma [18] are all impacted by a diagnosis of prostate cancer. Particularly, in a culture fraught with competition advocating, the possibility of public exposure persistently lingers in the background of their struggles with sexual impairment. A qualitative study [10] explored men’s experiences of prostate cancer and related stigma from a social-ecological perspective and reported sources of stigma from multiple levels, including the intrapersonal domain, such as poorer self-concept and identity due to sexual dysfunction and incontinence, the interpersonal or community domain, such as being reluctant to talk about cancer, and the institutional or public policy domain, such as poor communication and unmet psychosocial support from their physician.

Psychological distress is an important barrier to physical and mental health, and is underrecognized in prostate cancer care [19]. Notably, depression and anxiety are experienced as frequent and focal disruptions in both the general population and cancer patients. A meta-analysis [20] demonstrated that depression and anxiety were associated with a significant risk of higher cancer incidence, poorer cancer survival, and higher cancer-specific mortality. Compared with males with no lifetime history of any form of cancer, survivors of prostate cancer were 2.45 or 2.05 times more likely to screen positive for current anxiety or depressive symptoms, respectively [21]. The diagnosis and treatment of cancer is a stressful experience, and illness uncertainty is a source of that stress. Another study [22] documented that patients’ illness uncertainty also has a direct and negative impact on their physical and mental well-being. In summary, it would appear that a significant proportion of males who had prostate cancer are troubled by psychological disorders, while the extent of the impact of anxiety and depression and uncertainty in illness on QoL remains less conclusive.

Some studies [21, 22] have examined QoL issues, stigma problems, or psychological disorders in prostate cancer. However, questions remain regarding the possible associations among them, apart from efforts to minimize their individual intrusion into patients’ daily lives. This study describes the associations between stigma, anxiety and depression, QoL, and uncertainty in illness in prostate cancer patients. Based on Goffman’s theory [11] that stigmatization is highly connected with mental health from a psychosocial perspective, it was hypothesized that, stigma would be related to poorer QoL (Hypothesis 1), higher levels of anxiety and depression (Hypothesis 2) and uncertainty in illness (Hypothesis 3), and that anxiety and depression would be negatively associated with QoL (Hypothesis 4). In view of the studies in the literature that highlight the mediating role of anxiety and depression [23, 24] in prostate cancer, and considering that stigmatization is highly linked to mental health (Goffman’s theory), an additional hypothesis (Hypothesis 5) was defined to study the indirect effects of anxiety and depression on the relationship between stigmatization and QoL. Since this study is cross-sectional, it is only possible to study the direct and indirect effects, and not the mediating role [25].

## Methods

### Participants and procedures

A cross-sectional design and convenience sampling method were used in this study. Patients hospitalized in any one of four urology wards were invited to participate in this study between October 2020 and April 2021. The inclusion criteria were as follows: (1) diagnosed with prostate cancer; (2) under treatment or having completed at least one treatment intervention, such as radical prostatectomy, androgen deprivation therapy, chemotherapy, radiotherapy, or active surveillance; (3) with the ability to talk and write; and (4) agreed to participate in this study. Patients who were not informed of their exact condition, were in an unstable condition or had serious complications were excluded. Informed written consent was obtained from all participants enrolled in the study. Participants were invited to complete a general information questionnaire on demographic information and clinical data, and self-assessment scales. Researchers would provide supplementary explanations if necessary. This study received approval from the Clinical Research Ethics Committee of the First Affiliated Hospital, Zhejiang University School of Medicine (Approval No. 2018–707).

In total, 263 participants were recruited in this study, with an average age of 68.17 (*SD* = 6.25) years. The participants reported their educational background as middle school or lower (51.7%), high school (31.6%), or college and upper (16.8%). The majority of the sample reported being in a marital relationship (90.1%), with 9.9% as other (single, divorced, separated, and widowed). The sample comprised 250 males with medical insurance (95.1%), 8 with free medical treatment (3%), and 5 at their own expense (1.9%). When asked about their self-care agency, 178 participants reported “Good” (67.7%), 67 reported “Not bad” (25.5%), and 18 reported “Poor” (6.8%). Of the 263 participants (prostate specific antigen *M* = 16.40, *SD* = 22.23), a large majority (98.9%) were treated with surgery, and 14 (5.3%) received radiotherapy. For the TNM classification of malignant tumors, most participants (46.8%) were in stage II, 33.8% were in stage III, 10.6% were in stage I, and were in 8.7% stage IV.

### Measures

#### Stigma

Stigma was assessed using Stigma Scale for Chronic Illness (SSCI), which is a 24-item measurement of stigma [26] encompassing two dimensions, named "self/internalized stigma" (13 items, range 1–13) and “enacted stigma” (11 items, range 14–24). Items are rated on a five-point Likert scale from 1 (never) to 5 (always). The scale was developed to better understand the impact of stigma on people across chronic illnesses with good internal consistency (Cronbach’s *α* = 0.97), convergent validity, and item response theory model fit.

#### Quality of life

The 26-item Chinese version of the World Health Organization Quality of Life assessment (WHOQOL-BREF) [27], which was used to measure participants’ QoL in the past four weeks includes four domains: physical health, psychological, social relationships, and environment. The WHOQOL-BREF domain scores demonstrate good discriminant validity, content validity, internal consistency, and test–retest reliability. Participants rate the extent to which they agreed with each statement on a five‐point scale from 1 to 5, with higher scores indicating better QoL.

#### Anxiety and depression

Subjects’ states of depression and anxiety were detected by the Hospital Anxiety and Depression Scale (HADS) [28]. The questionnaire consists of 14 items in total, half of which assess signs of anxiety (HADS-A), while the other half assess depression (HADS-D). The HADS has been widely used to identify hospitalized patients with anxiety and depression. This tool had been validated in previous studies as a reliable instrument for use in cancer patients [29–31]. The reliability of the HADS has been tested, with a Cronbach’s alpha of 0.86 for the full scale [32].

#### Uncertainty in illness

The Mishel Uncertainty in Illness Scale for Adults (MUIS-A) was developed by Mishel [33] to explore the role of uncertainty in affecting patients’ experiences in illness, treatment, and hospitalization, and it was introduced to China by Sheila Sheu [34]. The Chinese version of the MUIS-A is a 25-item questionnaire with two dimensions (ambiguity and complexity) answered on a five-point continuum ranging from 1 (strongly disagree) to 5 (strongly agree). This scale has been widely accepted as a valid screening tool for uncertainty in illness, with a Cronbach’s alpha coefficient of 0.865.

### Data analysis

All variables were checked for normality before analysis. First, IBM SPSS Statistics 24.0.0 was used for data entry and analysis. Subsequently, Pearson correlation analysis was performed between the main variables. Primary hypotheses were tested by structural equation modeling with IBM SPSS Amos (version 24.0.0), and a path analysis by bootstrapping was conducted [35].

## Results

### Preliminary analysis

Pearson correlation analysis for the main variables was displayed in Table 1. The correlations provided initial support for earlier hypotheses. These results indicated that stigma was negatively associated with QoL (*r* = − 0.251, *p* < 0.01); in contrast, it was positively associated with anxiety and depression (*r* = 0.229, *p* < 0.01), and uncertainty in illness (*r* = 0.150, *p* < 0.05). QoL was negatively associated with anxiety and depression (*r* = − 0.357, *p* < 0.01). Associations between uncertainty in illness and QoL and anxiety and depression were not significant ( *p* > 0.05).

**Table 1:** Pearson correlation analysis for the main variables (*N* = 263)

Variable(s)	*Mean* ± *SD*	1	2	3	4	5	6	7	8	9	10	11	12
1. **Stigma**	43.88 ± 13.55	--											
2. Internalized stigma	26.13 ± 8.38	0.917^**^	--										
3. Enacted stigma	17.75 ± 6.75	0.869^**^	0.599^**^	--									
4. **Quality of life**	49.61 ± 6.92	-0.251^**^	-0.230^**^	-0.219^**^	--								
5. Physical health	12.34 ± 2.09	-0.145^*^	-0.151^*^	-0.104	0.794^**^	--							
6. Psychological	12.35 ± 2.10	-0.168^**^	-0.146^*^	-0.155^*^	0.829^**^	0.587^**^	--						
7. Social relationships	12.46 ± 2.31	-0.224^**^	-0.213^**^	-0.185^**^	0.776^**^	0.463^**^	0.522^**^	--					
8. Environment	12.46 ± 2.21	-0.257^**^	-0.216^**^	-0.247^**^	0.789^**^	0.506^**^	0.551^**^	0.454^**^	--				
9. **Anxiety and Depression**	28.31 ± 7.58	0.229^**^	0.303^**^	0.084	-0.357^**^	-0.346^**^	-0.255^**^	-0.264^**^	-0.274^**^	--			
10. **Uncertainty in illness**	82.03 ± 9.05	0.150^*^	0.137^*^	0.132^*^	-0.058	-0.048	-0.054	0.041	-0.128^*^	0.087	--		
11. Ambiguity	49.06 ± 6.11	0.122^*^	0.108	0.110	-0.053	-0.058	-0.062	0.046	-0.101	0.074	0.955^**^	--	
12. Complexity	32.87 ± 3.66	0.138^*^	0.129^*^	0.117	-0.059	-0.039	-0.043	0.017	-0.125^*^	0.073	0.891^**^	0.761^**^	--

*Notes*: ^*^p < 0.05, ^**^p < 0.01

### Hypothesis testing

#### Model fit

Model fitness mainly comprises three parameters [36]: absolute fit measurement (such as *GFI*, *AGFI*, and *RMSEA*), incremental fit measurement (such as *NFI*, *IFI*, and *CFI*), and parsimonious fit measurement (such as *CN* and *AIC*). The *GFI*, *AGFI*, *NFI*, *IFI*, and *CFI* statistics should be at or above 0.90 [37, 38], while an *RMSEA* below 0.08 represents a good fit [39]. The *CN* should be at or above 200, and the *AIC* of the default model should be less than that of the saturated model as well as the independent model [38]. The proposed research model had good fit indices, as indicated in Table 2.

**Table 2:** Model fit

	Parameter(s)							
	Absolute fit measurement			Incremental fit measurement			Parsimonious fit measurement	
	*GFI*	*AGFI*	*RMSEA*	*NFI*	*IFI*	*CFI*	*CN*	*AIC*
**Model**	0.994	0.945	0.035	0.950	0.966	0.963	345	62.068 *Default model*
								65.722 *Saturated model*
								84.505 *Independence model*

Abbreviations: GFI, goodness-of-fit index. AGFI, adjusted goodness-of-fit index. RMSEA, root mean square error of approximation. NFI, normed fit index. IFI, incremental fit index. CFI, comparative fit index. CN, critical N. AIC, Akaike information criteria

**Table 3:** All direct paths of the model. (*N* = 263)

Predictor variable(s)	Outcome variable(s)	*B*	*S.E.*	*C.R.*	*β*	*P* value
Stigma	Quality of life	-5.603	1.655	-3.386	-0.209	0.001
Stigma	Anxiety and depression	0.445	0.203	2.189	0.135	0.001
Stigma	Uncertainty in illness	4.720	2.194	2.152	0.126	0.029
Anxiety and depression	Quality of life	-2.541	0.478	-5.319	-0.312	0.031
Uncertainty in illness	Quality of life	0.445	2.194	2.152	-0.039	0.524

**Fig. 1:**
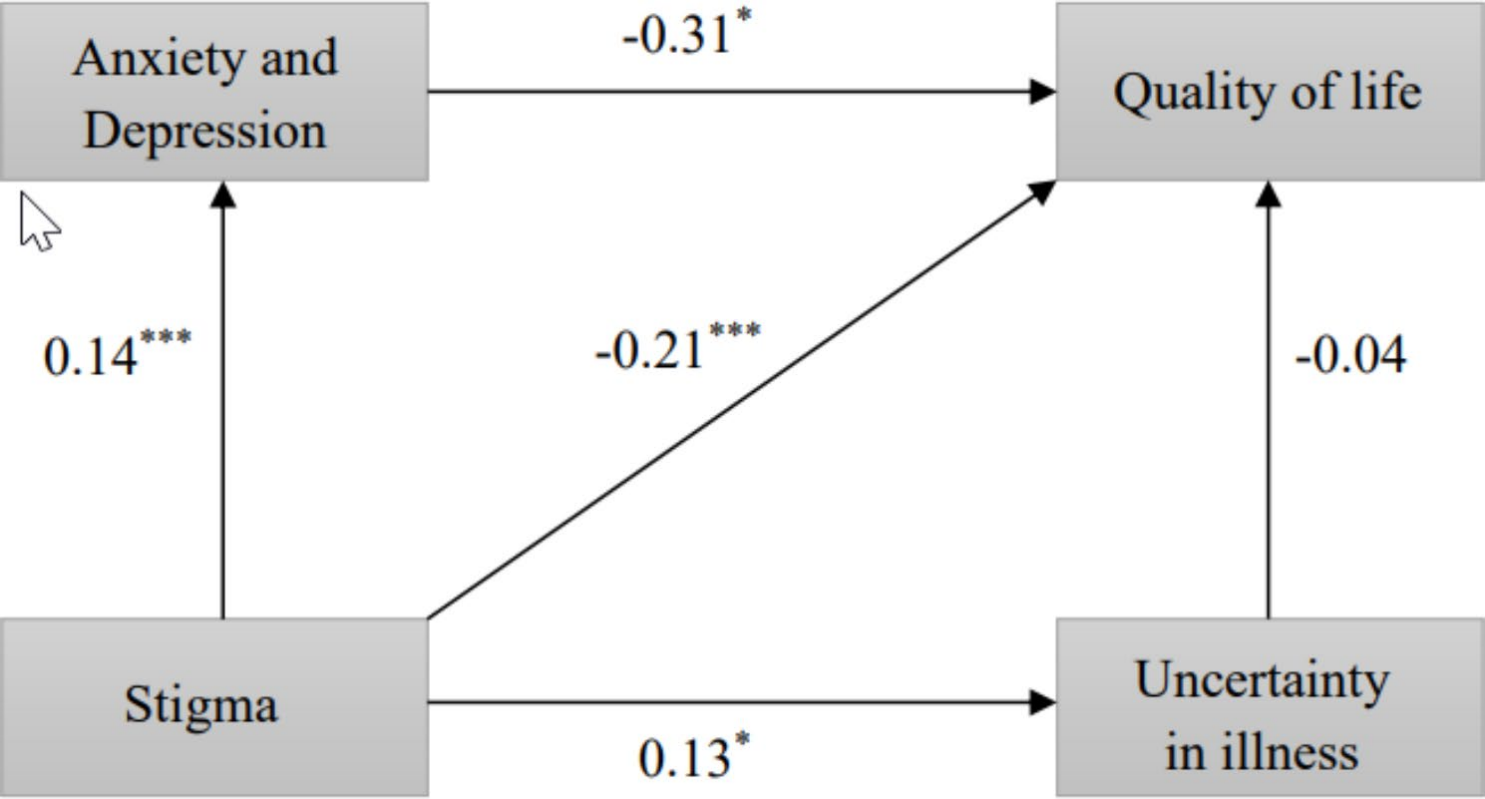
The research model for the relationship between stigma, anxiety and depression, uncertainty in illness and QoL. Abbreviations: QoL, quality of life. Notes: Standardized path coefficients are presented. ^*^p < 0.05, ^***^p < 0.001

#### Associations between stigma, anxiety and depression, QoL, and uncertainty in illness

The results of the path analysis are reported in Table 3. Fitting with research Hypothesis 1, stigma was significantly negatively related to QoL (*β*=−0.209, *S.E.* =1.655, *p* < 0.001). Moreover, stigma was positively related to anxiety and depression (*β* = 0.135, *S.E.* =0.203, *p* < 0.001) and uncertainty in illness (*β* = 0.126, *S.E.* = 2.194, *p* < 0.05), which provide support for Hypothesis 2 and 3. As hypothesized, anxiety and depression overall were significantly negatively related to QoL, with a standardized regression coefficient (*β*=−0.312, *S.E.* =0.478, *p* < 0.05); that is, participants reporting higher levels of anxiety and depression reported decreased levels of QoL, providing support for Hypothesis 4. In addition, the study showed that in the presence of a third variable (anxiety and depression overall), direct effects of stigma on QoL (β=−0.209, S.E. =1.655, p < 0.001) are reduced, as indirect effects emerge through the variable anxiety and depression overall, with an indirect effect size of − 0.054. A research model was established for the relationship between stigma, anxiety and depression, QoL, and uncertainty in illness, as depicted in Fig. 1.

## Discussion

This study was conducted by verifying that stigma affects prostate cancer patients with anxiety and depression, uncertainty in illness, and poor QoL. This study reinforces the implications that health care professionals should pay more attention to caring for patients’ mental illness, and strive to minimize levels of stigma, anxiety and depression, and uncertainty in illness to improve QoL outcomes.

Prostate cancer patients feel restrained by their disease. Most patients with prostate cancer are treated with prostatectomy, hormone therapy, or radiation treatment that consistently results in either reduced sexual desire, or the complete loss or attenuation of ejaculate [40]. All of these treatments have a strong likelihood of adversely affecting men’s sexual functioning, which is an indispensable part of QoL as well as a primary source of stigma. Patients with prostate cancer typically worry about the effect of physical limitation because of feelings of frustration or loss of dignity, whereas for partners, physical deterioration is the visible manifestation of the passage of their spouse toward incapacity and death [41], and these intensify perceived stigma levels. Conversely, with most prostate cancer patients in their 70s or above, elderly Chinese males are conservative regarding emotional expressiveness, especially unpleasant expressiveness, in public, which exacerbates their psychological distress and may negatively influence help-seeking behaviors and overall patient outcomes. With great economic improvements, Chinese people are eager to obtain a higher QoL. While happiness and satisfaction are important components of quality of life in Western culture, Chinese philosophies such as Confucianism, Buddhism, and Taoism virtuously uphold forbearance, humility and harmony [42]. Consequently, perceived stigma and psychological distress, such as anxiety and depression, break the balance for inner peace.

Many studies [10, 12, 15, 43–45] suggest that stigma affects mental health, such as anxiety and depression, and leads to poorer health-related QoL, although some studies were conducted using a qualitative approach [10, 44]. One study [45] assessed the relationships between stigma, as experienced by prostate cancer survivors, QoL, and relationship satisfaction for survivors and their spouses. However, the role of psychological distress was not examined, and only 80 participants, comprising prostate cancer survivors and their spouses, were included. The topics of stigma and anxiety about prostate cancer were discussed in another study [46], but they were described as mediators of doctors’ empathy abilities on the cellular immunity of patients. This study highlights that stigma is associated with anxiety and depression, and both have negative effects on QoL among prostate cancer patients. These findings confirmed the results of previous studies [15, 45], which argued that perceived stigma and psychological distress are recognized barriers to improve QoL. Attempts [47, 48] have been made in recent years to cope with mental illness, such as stigma, anxiety and depression, through the use of counseling services and open communication among prostate cancer patients. Given the complex nature of stigma and psychological distress as well as potential cultural issues [49], more extensive studies are warranted.

This paper enriched the literature on psychological aspects of prostate cancer by examining the association between stigma, anxietyand depression, uncertainty in illness, and QoL and may have implications for health care professionals. First, necessary patient education should be given high priority. Medical institutions or units are expected to cultivate an open and harmonious treatment atmosphere. Under these circumstances, patients are encouraged to express their inner feelings gradually shape a healthy understanding of diseases and improve health literacy. Necessary spaces and places should be provided for expressing and venting negative emotions. Small communities, such as prostate cancer support groups (PCSGs), where they share information and find social support, provide an important platform for information exchange and emotional support in the field of prostate cancer [48]. Second, more targeted psychological self-care strategies should be shared with patients. It has been reported [10] that patients who employ higher levels of some coping mechanisms, such as humor and venting, may find benefits in tackling stigma related to prostate cancer. Additionally, health care providers may be able to alleviate uncertainty in illness by providing patient navigation and improving information delivery, or providing psychosocial counseling to individuals experiencing feelings of excessive levels of anxiety and depression. Furthermore, to improve the health-related QoL of prostate cancer patients, it is critical that healthcare professionals encourage patients to maintain healthy emotions while advising early detection (PSA test) and promoting medication adherence. Research analyses suggest that psychological distress may have an etiological role and a prognostic impact on cancer [20]. Men with prostate cancer who experience symptoms of depression or anxiety should seek professional help early on, which was also implied in another investigation [43]. Further research is need to delineate other psychological factors that influence patients’ perceived stigma and health-related QoL. For a disease as common as prostate cancer, efforts to explore patients’ mental health issues and the provision of psychological help are critical for health promotion. The results from the current study support the important role that anxiety and depression have on patients’ perceived stigma and QoL. Future interventions should focus on educating patients about the importance of eliminating psychological distress, such as stigma, anxiety and depression, and uncertainty in illness, for health outcomes. Ideally, these interventions would benefit patients during recovery and ultimately help them achieve physical and mental health for their whole life.

However, several limitations should be considered. First, causality or mediation cannot be inferred in a cross-sectional design; thus, only the direct and indirect effects of the relationships were referred to in this study. Second, the study participants were recruited under strict inclusion and exclusion criteria, and the instruments have good validity and reliability; however, bias, such as selection bias and measurement bias, cannot be neglected. Another limitation of the study is the lack of supported data from a control group. Furthermore, only the overall role of anxiety and depression was analyzed in the proposed model, which should be explored separately in future studies. In addition, further research on demographic and clinical variables is warranted.

## Conclusion

This study investigated the associations between stigma, anxiety and depression, uncertainty in illness and QoL. The study findings emphasize the gravity of stigma as a psychosocial concern. Health care professionals may help patients alleviate feelings of anxiety and depression, and uncertainty in illness to improve QoL outcomes.

## Data Availability

All data that support the findings of this study are available on request from the corresponding author. The data are not publicly available due to privacy or ethical restrictions.
